# Should I have this on my bookshelf? A review of Cardiac Drugs in Pregnancy

**DOI:** 10.3389/fphar.2014.00249

**Published:** 2014-11-12

**Authors:** Michael J. Rieder

**Affiliations:** Departments of Paediatrics, Physiology and Pharmacology and Medicine, Schulich School of Medicine and Dentistry, Western UniversityLondon, ON, Canada

**Keywords:** cardiovascular drugs, pregnancy, hypertension, heart failure, arrhythmias

“Cardiac drugs in pregnancy” is a pocket-sized book published by Springer as part of their “Current Cardiovascular Therapy” series. The book is a 130 page synopsis of cardiovascular therapy in the setting of pregnancy, edited by two South African cardiologists, Drs. Karen Sliwa and John Anthony. There are six chapters in the book, beginning with General Principles and then proceeding through a series of common cardiovascular problems such as hypertension, heart failure, and arrhythmias written by a series of experienced South African and European cardiologists.

The title and topic seem unusual at first glance—after all, how common is cardiovascular therapy in pregnancy? The editors make the case—and this reviewer agrees with them—that cardiovascular disease is pregnancy is in fact relatively common, notably in the era of older and older women having children, and that cardiologists need an approach to these problems. As well, some of the few well-described human teratogens include drugs commonly used by cardiologists, notably the angiotensin-converting inhibitors and warfarin. The book is an easy and enjoyable read, clearly written, and intended for practicing clinicians. The editors and chapter authors have focused on the common and important rather than the esoteric and arcane, which this reviewer thoroughly agrees with. As an example, fetal therapy is not discussed, being beyond the scope of this book. The first chapter deals with how the cardiovascular system adapts during pregnancy and how this may impact on therapy. Succeeding chapters are disorder-specific. The authors take care to ensure that their chapters provide not only a conceptual overview but also recommendations to guide therapy. The authors address what is confirmed by the literature and, of equal importance, areas where there is paucity of evidence based medicine (or data) to guide therapy decisions of cardiovascular disease in pregnancy.

That being said, the book is not perfect and one wonders if perhaps the inclusion of one of small but active community of human teratogen investigators or a consulting obstetrician as a chapter author might have been useful. As an example, the concept of baseline risk and the importance of a fulsome discussion on baseline risk as part of the evaluation and therapeutic planning for pregnancy is understated, which is regrettable given the clear and compelling evidence as to the importance of this—and the consequences of misinformation—in the setting of pregnancy and drug therapy. There is also a somewhat uncritical view of some important issues in the care of the pregnant woman. As an example, more than one chapter cites the US Food and Drug Administration classification of drug teratogenicity in terms of assessment of pregnancy risk without a deeper discussion as to the very real limitations—acknowledged by the FDA - of this classification system in terms of real world evidence. The chapter on cardiovascular adjustments during pregnancy is there but is somewhat light on some issues such as drug transfer across the placenta, which is an increasingly interesting and complex area of study. An increasing population of cardiac patients—adult survivors of congenital cardiac disease—is not covered in a separate chapter but is dealt with piece-meal. It seems to this reviewer that this is not the best way to address the complex problems of a new and growing population of cardiac patients, and the addition of a chapter on pregnancy in adult survivors of congenital cardiac disease would be a useful addition to the next edition of this book.

These reservations aside, the book does bring together in a single small volume a large amount of highly relevant and practical material covering those conditions and problems most likely to be encountered by cardiologists and consulting internists. The advantages and limitations of current therapy are concisely outlined and the recommendations provided will be very useful in guiding therapy. The inclusion of a final chapter on the use of obstetrical drugs in the management of cardiovascular disease is both novel and very interesting.

In summary, this small volume is a useful and practical book that would be a very useful part of the library of cardiologists as well as internists whose consulting practices include the care of pregnant women.

## Conflict of interest statement

The author declares that the research was conducted in the absence of any commercial or financial relationships that could be construed as a potential conflict of interest.

